# Family environment and polygenic risk in the bipolar high‐risk context

**DOI:** 10.1002/jcv2.12143

**Published:** 2023-03-16

**Authors:** Emma K. Stapp, Janice M. Fullerton, Rashelle J. Musci, Peter P. Zandi, Melvin G. McInnis, Philip B. Mitchell, Leslie A. Hulvershorn, Neera Ghaziuddin, Gloria Roberts, Alessandra G. Ferrera, John I. Nurnberger, Holly C. Wilcox

**Affiliations:** ^1^ Department of Mental Health Johns Hopkins Bloomberg School of Public Health Baltimore Maryland USA; ^2^ Genetic Epidemiology Branch National Institute of Mental Health Bethesda Maryland USA; ^3^ Neuroscience Research Australia Randwick New South Wales Australia; ^4^ School of Medical Sciences University of New South Wales Sydney New South Wales Australia; ^5^ Department of Psychiatry University of Michigan Ann Arbor Michigan USA; ^6^ School of Psychiatry University of New South Wales Sydney New South Wales Australia; ^7^ Department of Psychiatry Indiana University School of Medicine Indianapolis Indiana USA; ^8^ Department of Medical and Molecular Genetics Indiana University School of Medicine Indianapolis Indiana USA; ^9^ Stark Neurosciences Research Institute Indiana University School of Medicine Indianapolis Indiana USA

**Keywords:** attempted, bipolar disorder, gene‐environment interaction, multifactorial inheritance, parent‐child relations, suicide

## Abstract

**Background:**

The interaction of polygenic risk (PRS) and environmental effects on development of bipolar disorder (BD) is understudied, as are high‐risk offspring perceptions of their family environment (FE). We tested the association of offspring‐perceived FE in interaction with BD‐PRS on liability for BD in offspring at high or low familial risk for BD.

**Methods:**

Offspring of a parent with BD (oBD; *n* = 266) or no psychiatric disorders (*n* = 174), aged 12–21 at recruitment, participated in the US and Australia. Empirically‐derived profiles of FE classified offspring by their perceived levels of familial cohesion, flexibility, and conflict. Offspring BD‐PRS were derived from Psychiatric Genomics Consortium BD‐GWAS. Lifetime DSM‐IV bipolar disorders were derived from the Schedule for Affective Disorders and Schizophrenia for School‐Aged Children. We used a novel stepwise approach for latent class modeling with predictors and distal outcomes.

**Results:**

Fifty‐two offspring were diagnosed with BD. For those with well‐functioning FE (two‐thirds of the sample), higher BD‐PRS tracked positively with liability for BD. However, for those with high‐conflict FEs, the relationship between BD‐PRS and liability to BD was negative, with highest risk for BD observed with lower BD‐PRS. In exploratory analyses, European‐ancestry offspring with BD had elevated history of suicidal ideation in high‐conflict FE compared to well‐functioning‐FE, and of suicide attempt with low‐BD‐PRS and high‐conflict FE.

**Conclusions:**

The data suggest that the relationship of BD‐PRS and offspring liability for BD differed between well‐functioning versus high‐conflict FE, potentially in line with a multifactorial liability threshold model and supporting future study of and interventions improving family dynamics.

## Graphical abstract

We examined the joint contribution of offspring‐perceived family environment and bipolar‐polygenic risk score to the odds of bipolar diagnosis and related clinical features in adolescents at high or low familial risk for bipolar disorder. Findings may suggest different ways of crossing the liability threshold to develop early‐onset bipolar disorder, consistent with a multifactorial liability threshold model. Namely, offspring with lower bipolar‐polygenic risk score, in the presence of interpersonal environmental risk (conflicted family environments), had higher odds of bipolar diagnosis and greater prevalence of suicide attempt, in contrast to offspring with well‐functioning family environment.

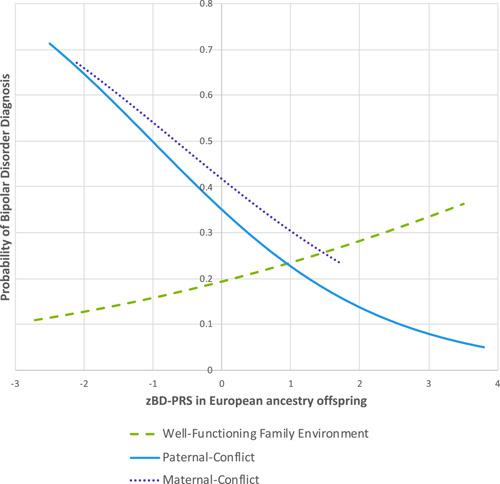

**Key points**

- Family history is the strongest known predictor of bipolar disorder (BD), with genetics and family environment (FE) being key influences on high‐risk offspring.
- We demonstrate that FE and polygenic risk for BD (BD‐PRS) interact to confer liability for BD, especially among offspring identifying paternal‐conflict compared to well‐functioning FE.
- Affected offspring with low‐BD‐PRS/high‐conflict‐FE had increased suicidal ideation and attempt
- A small but converging body of evidence is consistent with the liability threshold model, whereby lower polygenic burden in the presence of interpersonal environmental risk (i.e., multifactorial) is associated with increased mood disorder liability; replication is needed.
- Clinically, these results support focusing on modifiable domains of FE, such as reducing communication conflict and improving family cohesion and adaptability.

Decades of genetic inquiry have demonstrated that bipolar disorder (BD) is a complex disorder with genetic and environmental risk factors, and family history remains its strongest known predictor (Craddock & Sklar, 2013). One model that has been proposed for inheritance of complex disorders is the multifactorial liability threshold model. The assumption, building on diathesis‐stress models, is that liability for a disorder is a continuum, and that when an individual's combined liability from multiple factors crosses an unobserved threshold, he or she will develop the disorder (Gottesman & Shields, 1967; McGue et al., 1983). Genetic susceptibility may vary for an individual depending on gene expression and the environment to which they are exposed. However, disambiguation of the relative effects of genes and environment in family studies is challenging, and there is little literature on polygenic‐environmental interaction effects on liability (Uher & Zwicker, 2017; Visscher & Wray, 2015).

Genetics have been shown to play a substantial role in conferring risk for BD (Craddock & Sklar, 2013; Mullins et al., 2021; Stahl et al., 2019). Common single‐nucleotide polymorphism (SNP) variants associated with BD are of small individual effect at the population‐level, but they additively increase risk and together are estimated to account for 17%–23% of the genetic variance in risk for BD (Mullins et al., 2021; Stahl et al., 2019). In turn, these BD‐associated SNPs may be used to create a polygenic risk score (PRS), which may be used as a measure of genetic burden (Fullerton & Nurnberger, 2019; Wray et al., 2014), although PRS alone are not sufficiently informative to predict future disorders. While PRS have their limitations—explaining 4%–5% of the phenotypic variance on the liability scale for BD (Mullins et al., 2021; Stahl et al., 2019) and typically ignoring higher‐order gene‐gene and gene‐environment interactions and other classes of genetic variation—they can be a useful tool to quantify genomic risk load and to explore relationships with other risk factors.

Among non‐genetic influences, family environment (FE) is pivotal. Parental care has been shown to influence brain and neuroendocrine development including stress responsivity, and excessive interpersonal stress has been implicated in the onset, recurrence, severity, and excess morbidity associated with BD (Hodgins et al., 2002; Lippard & Nemeroff, 2020; Miklowitz & Chung, 2016; Post & Leverich, 2006; Shakiba et al., 2020). Offspring of BD parents are at 8–10 fold increased risk of developing BD (Craddock & Sklar, 2013) and overall increased risk of developing mood and psychiatric disorders (Hodgins et al., 2002; Rasic et al., 2014) compared to offspring of parents without psychiatric disorders, yet most high‐risk offspring do not themselves develop BD. Thus parent‐child relationships, and FE more generally, may elucidate environmental influences on risk of psychopathology, and modifiable targets for prevention. Findings from a systematic review of prospectively‐measured FE and offspring psychiatric disorders demonstrated that parents with BD report lower family cohesion than parents without psychiatric disorders, as well as higher conflict when offspring have BD rather than being unaffected, however offspring perceptions of the FE have been understudied (Stapp, Mendelson et al., 2020). We sought to clarify these relationships in a multi‐site collaborative study of adolescent and emerging adult offspring of parents with BD and of controls, by testing the relationship of offspring‐perceived FE in interaction with BD‐PRS on liability for offspring BD.

## METHODS

### Participants and procedures

The study sample consists of 440 participants in the US and Australia aged 12–21 years at time of recruitment from 2006 to 2013 into a prospective study of adolescents at high (*n* = 266) or low (*n* = 174) familial risk for BD (Nurnberger et al., 2011; Perich et al., 2015). Offspring at familial high‐risk for BD were identified through probands with DSM‐IV bipolar I disorder (BD‐I), bipolar II disorder (BD‐II), or schizoaffective disorder bipolar type (SAB) from the NIMH Genetics Initiative bipolar sample and other genetic studies, specialty clinics, and the general public. Control participants were recruited from general practitioners, motor vehicle records, and advertising, excluding individuals with a parent or sibling with BD‐I, BD‐II, recurrent major depression (MDD), schizoaffective disorder, schizophrenia, recurrent substance abuse, or any psychiatric hospitalizations, or whose parent had a first‐degree relative with a history of psychosis or hospitalization for a mood disorder. Parent diagnostic status was confirmed using the Diagnostic Interview for Genetic Studies (Nurnberger et al., 1994). Although participants in the primary study also included siblings and second‐degree relatives of BD probands, the current analysis focuses specifically on offspring (“oBD”) because of our interest in the parent‐child relationship. In some families, multiple offspring participated, and this was accounted for in analyses.

#### Ethical considerations

Institutional Review Boards approved the research at each of the four US sites, and the University of New South Wales Human Research Ethics Committee approved the research at the Australian site. Written informed consent was obtained for all adult participants, or assent with parental consent for participants under age 18 in the US and 17 in Australia, after receiving a complete description of the study.

### Measures

#### Outcome: Offspring bipolar disorder

Offspring were interviewed by extensively trained clinicians using the *Schedule for Affective Disorders and Schizophrenia for School‐Aged Children*, *bipolar disorder version* (K‐SADS‐BP) (Nurnberger et al., 2011). Lifetime DSM‐IV disorders were confirmed by best‐estimate consensus of two clinicians using direct interviews of offspring and parents and medical history records. A binary primary outcome variable of BD was created using all available information, which included BD‐I, SAB, BD‐II, and BD not otherwise specified (BD‐NOS) subtypes. Generally BD‐NOS was diagnosed only if the participant approached criteria for BD‐II but had one fewer symptom in hypomanic and depressive categories; diagnoses of mania were made by consensus, with strict adherence to DSM‐IV criteria (Nurnberger et al., 2011). Diagnoses were available for 93% of the offspring.

#### Exposure: Family environment profiles

We previously identified three latent profiles of offspring‐perceived FE (Stapp et al., 2019), accounting for within‐family clustering. The FE profiles were derived from children's reports on conflict with their mothers and fathers using the *Conflict Behavior Questionnaire* (CBQ) (Prinz et al., 1979); family adaptability and cohesion subscales using the Family Adaptability and Cohesion Evaluation Scales, version II (FACES‐II) (Olson et al., 1982); and factor scores for maternal warm engagement and permissiveness from the *Home Environment Interview for Children* (HEIC) (Reich & Earls, 1984). For the HEIC, youth reported on the past year if currently living with their biological parent(s), or the last year they lived together if currently living apart. For the CBQ and FACES, the timeframe for describing family or relationships was current at assessment, without an exact period specified. High‐risk and control offspring were modeled together and present in all FE profiles. The largest group of offspring (67.7%) perceived FE characterized by nurturance, flexibility, and low conflict (“well‐functioning”), whereas the two smaller profiles were characterized by low warmth and cohesion, rigidity, and high conflict. Of the latter, a medium‐sized group (20.8%) clustered together based on high conflict with father and low family cohesion and flexibility (“paternal‐conflict”), and the smallest group (11.5%) clustered together based on very high conflict and rigidity in the mother‐child relationship (“maternal‐conflict”).

#### Exposure: Genetic risk

##### Genotyping

DNA extraction and genotyping of the cohort has been described elsewhere (Fullerton et al., 2015; Wilcox et al., 2017). Peripheral blood samples were collected from offspring for DNA extraction, and genome‐wide SNP genotyping was conducted using the Infinium PsychArray BeadChip (Illumina). Standard PGC pipelines were employed for genotype calling and quality control, and successfully genotyped SNPs had a pass rate of 99.6%. Genotype imputation procedures are detailed in the Supplemental Methods in the Supporting Information S1.

Principal component analysis (PCA) was performed in PLINK v1.90 (Purcell et al., 2007) using 164,680 independent SNPs, and genetic ancestry was defined based on the first two principal components (C1 and C2). Individuals with C1 and C2 values within one standard deviation (SD) of the mean of 1000 Genomes reference populations (EUR, EAS, AFR) were categorized as originating from those populations, whereas participants with PCA values between those defined groups were described as mixed‐ancestry. Genotype‐derived ancestry was available for 91% of the sample, with the genotyped sample being predominantly European (82%; Table 1).

**TABLE 1:** Sample characteristics for offspring in the bipolar high‐risk study.

	Total sample (*n* = 440)	High‐risk (*n* = 266)	Controls (*n* = 174)
Age, mean years ± SD	16.74 ± 2.85	16.59 ± 2.84	16.96 ± 2.87
Sex, *n* (%)			
Male	226 (51.36)	136 (51.13)	90 (51.72)
Female	214 (48.64)	130 (48.87)	84 (48.28)
Country, *n* (%)			
United States	320 (72.73)	194 (72.93)	126 (72.41)
Australia	120 (27.27)	72 (27.07)	48 (27.59)
Self‐reported race, *n* (%)			
White	392 (89.09)	243 (91.35)	149 (85.63)
Non‐white	48 (10.91)	23 (8.65)	25 (14.37)
PCA‐derived ancestry, *n* (%)	*n* = 399	*n* = 244	*n* = 155
European	327 (81.95)	218 (89.34)	109 (70.32)
Mixed Asian	19 (4.76)	9 (3.69)	10 (6.45)
Asian	8 (2.01)	0 (0)	8 (5.16)
Mixed African	12 (3.01)	10 (4.10)	2 (1.29)
African	33 (8.27)	7 (2.87)	26 (16.77)
Bipolar polygenic risk score	*n* = 399	*n* = 244	*n* = 155
zBD‐PRS, mean ± SD	1.82e−09 ± 1	−0.17017 ± 0.72081	0.26788 ± 1.2831
By PCA‐derived ancestry			
European	−0.35959 ± 0.2015069	−0.3613794 ± 0.187475	−0.3560112 ± 0.2278782
Mixed Asian	−0.1487443 ± 0.3721557	−0.0714711 ± 0.3270439	−0.2182901 ± 0.4130146
Asian	0.3340818 ± 0.1202569	–	0.3340818 ± 0.1202569
Mixed African	1.403639 ± 0.5593152	1.587564 ± 0.3955563	0.4840152 ± 0.0573329
African	3.057447 ± 0.3064666	3.146703 ± 0.2577897	3.033417 ± 0.3184954
	*n* = 410	*n* = 250	*n* = 160
Bipolar disorders, *n* (%)	52 (12.68)	47 (18.80)	5 (3.12)
Bipolar‐I	13 (25.00)	12 (25.53)	1 (20.00)
Schizoaffective disorder bipolar type	1 (1.92)	1 (2.13)	0 (0)
Bipolar‐II	17 (32.69)	15 (31.91)	2 (40.00)
Bipolar not otherwise specified	21 (40.38)	19 (40.43)	2 (40.00)

*Note*: BD‐PRS, bipolar disorder polygenic risk score based on disease associated SNPs from Psychiatric Genomics Consortium Wave 2 (Stahl et al., 2019) at *p*‐value threshold <0.03. Percentages are within column.

Abbreviations: PCA, principal components analysis; SD, standard deviation.

##### Bipolar disorder polygenic risk scores (BD‐PRS)

Disease‐associated SNPs and effect sizes were obtained from the PGC2‐BD discovery sample (Stahl et al., 2019), after excluding cohorts known to contain relatives of this study's participants (i.e., BMAU, GAIN, MICH, FAT2), in inverse‐variance weighted meta‐analysis using METAL (Willer et al., 2010), retaining 18,106 BD cases and 27,403 controls of European descent. PRSice v2 software (Choi et al., 2020) was used to create the additive PRS score for independent SNPs, weighted by the log odds ratio of disease‐association. The *p*‐value threshold that best distinguished BD‐status in a larger European‐ancestry adult cohort (*n* = 216 BD cases vs. *n* = 120 controls) was *p*_*T*_ < 0.03, which included 60,993 SNPs (*R* ^2^ = 0.046, *p* = 8.35e−05). Genotype data were available for 91% of the offspring. The BD‐PRS was standardized separately within the European‐ancestry subgroup for main latent analyses presented herein, and in the full offspring sample for sensitivity analyses (“diverse‐ancestry” models; see Supporting Information S1). We controlled for offspring genotype‐derived ancestry using PCA C1 and C2 in all models, as well as categorical PCA‐derived ancestry (European, Mixed Asian, Asian, Mixed African, and African) in diverse‐ancestry models to account for differences in linkage disequilibrium structures, in addition to genetic admixture within and across ancestries.

### Statistical analysis

We performed latent modeling in Mplus version 8.7 (Muthén & Muthén, 1998‐2017), using full information maximum likelihood and accounting for familial clustering. Sample statistics and regression using generalized estimating equations (GEE) to account for sibling relatedness were calculated using Stata Version 15 (StataCorp, 2017). Data on self‐reported demographic characteristics were complete (Table 1).

To test effects of FE and BD‐PRS on offspring BD, we used a stepwise approach for latent class modeling with predictors and distal outcomes (Masyn, 2017). This approach builds on the manual “BCH” method (Asparouhov, 2014) for auxiliary outcomes in Mplus. In contrast to one‐step approaches, this three‐step approach adjusts for covariate effects on both the categorical latent classes (exposure) and dichotomous outcomes. After the final unconditional latent profile analysis model is specified, individuals are classified into their most likely profiles using posterior probabilities and classification errors are calculated (modal classification). Then, the modal latent profiles with fixed classification errors are regressed on covariates and distal outcomes, adjusting for the effects of those covariates on the distal outcomes (Masyn, 2017) (see Figure S1).

We conducted Wald and pairwise tests of model significance for differences in prevalence of offspring BD across FE profiles, adjusting for the influence of age, sex, genetic ancestry, and BD‐PRS. Interaction models additionally tested associations of within‐profile BD‐PRS on BD. Models adjusting for site (US vs. Australia), self‐reported race, or family history of parental BD were not appreciably different in patterns and directionality of findings, and these were excluded from main analyses. Alpha was set at 0.05 using two‐tailed tests. Analyses presented in the main text were run in European‐ancestry offspring (*n* = 327) using BD‐PRS standardized within European‐ancestry offspring to ensure results were not influenced by population stratification, and parallel analyses in the full diverse‐ancestry sample are presented in full in the Supporting Information S1 and briefly discussed herein.

#### Post‐hoc analyses of clinical features

In European‐ancestry offspring with BD, we explored prevalence of baseline history of DSM‐IV alcohol or drug abuse or dependence (Hulvershorn et al., 2017) and lifetime suicide ideation or attempt (Wilcox et al., 2017) by their FE and/or BD‐PRS exposure. Offspring were dichotomized based on BD‐PRS as high (top two quintiles) or low (bottom three quintiles) (see Figure S2); in dichotomizing by BD‐PRS, we conducted these analyses in European‐ancestry only to avoid potential population stratification (see Figure S3 for graphical BD‐PRS distributions in each of 5 genetic ancestry categories). Offspring were assigned to their most likely FE profile (“hard classifying”) in Mplus, and then dichotomized as well‐functioning or “high‐conflict” (combining paternal‐ and maternal‐conflict profiles) in Stata. Frequency of clinical features were compared across combinations of exposures and tested using Pearson's chi‐square (all offspring with BD were unrelated, thus GEE was not used).

## RESULTS

### Sample characteristics

The study sample included 440 participants (representing 292 families) modeled together: 266 oBD and 174 controls (Table 1). Participants ranged in age from 12 to 22 years at time of initial interview (mean = 16.74, SD = 2.85), approximately half were male, and oBD and controls did not differ significantly on age, sex, or self‐reported race. Fifty‐two offspring received a diagnosis of BD (47 oBD, 5 control). The Supporting Information S1 contains mean BD‐PRS stratified by parent group (*p* = 0.281) and offspring affected status (see Table S1).

### Effects of family environment and BD‐PRS on offspring bipolar disorder

In the gene‐environment interaction model (Table 2, right), 11.8% of offspring with well‐functioning FE, 22.5% with paternal‐conflict, and 18.6% with maternal‐conflict were diagnosed with BD (*Wald* = 4.290, *p* = 0.117). A notably larger proportion of offspring with paternal‐conflict had BD compared to offspring with well‐functioning FE (*z* = 0.857, *p* = 0.055).

**TABLE 2:** Family environment and its interaction with bipolar polygenic risk score on offspring bipolar disorders in European‐ancestry offspring.

	Model Significance tests		
Family environment profile	Overall *Wald* and pairwise *z*		Proportion (*n*) with diagnosis
	Main effect of FE	BD‐PRS × FE interaction	
	*Wald* = 3.117, *p* = 0.2104	*Wald* = 4.290, *p* = 0.1171	
Well‐Functioning	–	–	0.118 (26)
Paternal Conflict	*z* = 0.796, *p* = 0.112	*z* = 0.857, *p* = **0.055** ^a^	0.225 (15)
Maternal Conflict	*z* = 0.752, *p* = 0.198	*z* = 0.748, *p* = 0.300^a^	0.186 (7)

*Note*: BD‐PRS based on disease associated SNPs from Psychiatric Genomics Consortium Wave 2 (Stahl et al., 2019) at *p*‐value threshold *p* < 0.03 standardized in European‐ancestry offspring only. Main effects model adjusted for offspring age, sex, genetic ancestry (first two components of continuous PCA‐derived genetic ancestry), and BD‐PRS. Interaction models adjusted for offspring age, sex, and genetic ancestry. *N*'s are estimates based on posterior probabilities rounded to the *nearest whole number*.

Abbreviations: BD‐PRS, bipolar polygenic risk score; FE, family environment; PCA, principal component analysis.

^a^ Statistical test of the significance of the interaction term of specific FE class with mean BD‐PRS.

**FIGURE 1:**
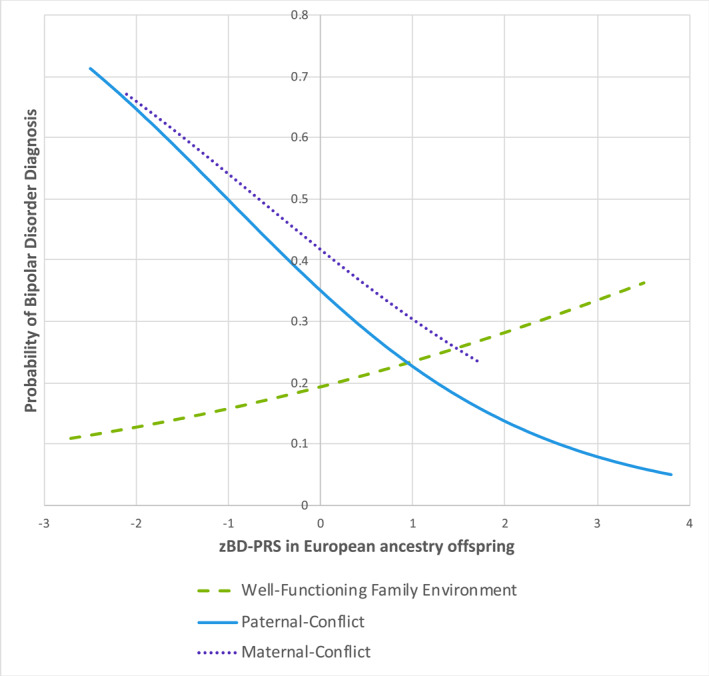
Estimated probability of bipolar disorder diagnosis by zBD‐PRS across three latent profiles of family environment in European‐ancestry offspring, adjusted for age, sex, and genetic ancestry. BD‐PRS, bipolar polygenic risk score.

Directionality between BD‐PRS and BD among offspring with well‐functioning FE (odds ratio [OR] = 1.28, 95% confidence interval [CI] = 0.80–2.05, *p* = 0.302) differed from those with paternal‐conflict, in whom a 1SD increase in BD‐PRS was marginally inversely associated with offspring BD (OR = 0.54, 95% CI = 0.27–1.11, *p* = 0.093); for maternal‐conflict the effect was similar though not significant (OR = 0.61, 95% CI = 0.16–2.28, *p* = 0.458). Thus, with lower BD‐PRS, highest risk for BD was among those with high‐conflict FEs, in stark contrast to those with well‐functioning FE (Figure 1).

### Sensitivity analyses

In diverse‐ancestry offspring, general patterns and directionality persisted (see Table S2 and Figure S4). As would be expected given the increase in sample size, effects were stronger for the overall gene‐environment interaction (*Wald* = 8.118; *p* = 0.017) and between BD‐PRS and paternal‐conflict compared to well‐functioning FE (*z* = 5.013, *p* = 0.011) on offspring BD. Additionally, interaction analyses in European‐ancestry offspring further adjusted for self‐reported race and parental BD are displayed in Figure S5, with attenuation of liability among offspring with well‐functioning FE but overall similar findings.

### Clinical features of offspring BD

In European‐ancestry offspring with BD, we observed a pattern of significantly elevated prevalence of suicidal ideation in those with high‐conflict‐FE compared to well‐functioning‐FE, irrespective of BD‐PRS (see Table S3). We also found a pattern of significantly elevated suicide attempt in those with low‐BD‐PRS, which was more pronounced in the presence of high‐conflict‐FE but also elevated in the context of well‐functioning‐FE (see Table S3). History of SUDs was not different across FE/BD‐PRS groups.

## DISCUSSION

In offspring at high or low familial risk for BD, our data suggest that family environment and polygenic risk for BD interact to increase liability for offspring BD. Importantly, BD‐PRS tracked inversely with liability for BD among offspring identifying high‐conflict FE, particularly paternal‐conflict (low cohesion and flexibility, high father‐child conflict), in contrast to offspring reporting well‐functioning FE (warm, flexible, low conflict). Additionally, among European‐ancestry offspring with BD, suicidal ideation was elevated in those with high‐conflict‐FE, and suicide attempt was elevated in those with lower BD‐PRS.

Our finding of a negative interaction between FE and BD‐PRS is consistent with the report of a negative gene‐environment interaction between MDD‐PRS and history of childhood trauma on depression. Mullins and colleagues (Mullins et al., 2016) found that individuals with depression and a history of moderate or severe childhood trauma tended to have lower PRS than other cases or controls, and suggested that problematic environmental exposure (childhood trauma) may be more important in the development of depression among individuals with lower genetic risk than for those with higher genetic risk. A subsequent meta‐analysis suggested trauma‐genetic interactions in the depression literature may have been due to chance, although re‐analysis of the Mullins sample individually using a larger discovery sample for PRS confirmed the original findings (Peyrot et al., 2018). Additionally, in over 400 adults with BD, higher reported levels of childhood emotional abuse were associated with lower BD‐PRS, and an interaction was observed such that patients with both higher total maltreatment and lower BD‐PRS presented with a higher risk of rapid cycling (Aas et al., 2020). Our multi‐measure construct of FE focused on familial climate overall, not limited to abuse, per se—the latter of which is at the extreme negative end of caregiving behaviors. That being said, some youth in our study who experienced high levels of conflict, rigidity, and low warmth may conceivably be experiencing FE that overlaps with maltreatment, which has been shown to have detrimental effects on health over the life course (Lippard & Nemeroff, 2020; Palmier‐Claus et al., 2016; Stapp, Williams et al., 2020). Taken together, a small but converging body of evidence is consistent with the liability threshold model, in which lower genetic burden, in the presence of environmental stress (i.e., multifactorial), is associated with increased mood and psychotic disorder liability (Schick et al., 2022; Tonini et al., 2022). These findings are also somewhat consistent with studies demonstrating the importance of early adversity and parent‐child relationship quality on differential stress responsivity and mood (Rudolph et al., 2020; Shakiba et al., 2020). Further research, including independent replication, is needed to elucidate the role of polygenic risk with interpersonal environmental impacts on youth development and psychopathology (Uher & Zwicker, 2017).

The modest associations we observed may be due to the relatively small number of offspring with BD, including a nontrivial number with well‐functioning FE. Maternal‐conflict was not significantly associated with offspring BD, possibly due to low representation of this FE corresponding with few diagnosed youth, although the pattern of association aligned with paternal‐conflict. Our sample had not fully passed the mean age of onset for BD in the population (Merikangas et al., 2011), warranting future clinical follow‐up. Early‐onset BD, such as is captured herein, is associated with poorer prognosis and clinical correlates compared to adult‐onset BD (Perlis et al., 2004). Consistent with this, we found that European‐ancestry offspring with BD with high‐conflict‐FE had elevated history of suicidal ideation compared to well‐functioning‐FE, suggesting a potential buffering effect of positive FE. Additionally, while those with low‐BD‐PRS had higher prevalence of suicide attempt in the presence of high‐conflict‐FE, suicide attempt was slightly less prevalent among those with well‐functioning‐FE. Though exploratory, our observations align with prior work highlighting the importance of severe environmental stressors on suicide attempt in those at risk for BD (Wilcox et al., 2017), and of poorer relationship quality with parents being longitudinally associated with increased risk of suicide ideation and attempt in youth with BD (Sewall et al., 2020).

The ability to identify features of the family environment, such as communication conflict, that are modifiable and to harness that knowledge for interventions that may prevent, lessen, or heal intergenerational risk processes is a public health priority (Raballo et al., 2021; Stapp, Mendelson et al., 2020). For example, family‐focused therapy (FFT) has been shown to hasten recovery and reduce mood symptom severity and recurrence in BD, particularly among families high in expressed emotion (high criticism, hostility, and enmeshment) (Miklowitz & Chung, 2016). In symptomatic youth at familial high‐risk for BD, FFT was associated with longer intervals to depression relapse (Miklowitz, Schneck et al., 2020) and without suicidal behaviors compared to enhanced care (Miklowitz, Merranko et al., 2020). Youth receiving FFT perceived significantly less conflict with their mothers, and family conflict significantly mediated the effects of treatment on suicide ideation at follow‐up, even when adjusting for current depression symptom severity. Our multi‐method measurement of FE, though not identical to expressed emotion, is easily obtained via offspring‐report.

By definition, our measure of genetic burden encompasses only common SNP‐based genetic variation, however different classes of genomic variation may contribute to BD risk and differentially mediate relationships with environmental factors. For example, BD is associated with increased rare variant burden (Goes et al., 2016), and epigenetic mechanisms likely mediate alterations in gene expression due to early life adversity (Lippard & Nemeroff, 2020). It is possible that common variants that are protective in a well‐functioning family may be vulnerability factors in a conflicted family. Likewise, there may be genetically‐influenced characteristics such as enhanced threat‐detection or altered perceptions thereof that are initially protective in high‐conflict environments (Shakiba et al., 2020). Disease‐associated SNPs used to create PRS are based on their main effect, but variants involved in gene‐environment interactions may underestimate effect sizes within specific environmental contexts, impacting predictive power of PRS in gene‐environment studies (Mullins et al., 2016); future research should seek to disentangle this. Further, most of our affected offspring had BD‐II or BD‐NOS, whereas our BD‐PRS was primarily derived from patients with BD‐I (Stahl et al., 2019). Separate risk scores for mania and bipolar depression or BD subtypes may therefore be informative, as there is mounting evidence that factors influencing BD‐I and BD‐II are different (Merikangas et al., 2014; Mullins et al., 2021; Song et al., 2018; Stahl et al., 2019; Vandeleur et al., 2014).

While we attempted to include a diverse genetic ancestry in this work, employing PCA‐derived ancestry variables in analyses, a primary limitation is the predominance of European‐ancestry participants. This limited our ability to conduct sensitivity analyses in non‐European‐ancestry participants and potentially limits generalizability, though patterns and directionality of findings from European‐ancestry analyses largely paralleled diverse‐ancestry analyses. This lack of diversity affects the entire field of psychiatric genetics, with transferability of European‐centric GWAS findings to other populations impacted by many factors (e.g., linkage disequilibrium, allele frequencies, genetic architecture); however, there is growing effort to increase diversity in large‐scale genomics discovery (Peterson et al., 2019). We also employed a *p*‐value threshold approach in generating PRS, but it is unclear exactly how many or which gene combinations may serve as a tipping point in the pathway of developing BD; the gene effects need not be the same in different individuals and gene interactions may not be additive (Visscher & Wray, 2015). Our family measures were self‐rating scales and not direct observations, which may provide alternative perspectives. Additionally, our FE was measured at a single time point, prohibiting causal attributions regarding directionality of FE and offspring mood. Future research should address timing of family function in relation to offspring BD due to reciprocal relations between family members. Finally, over one‐third of the offspring with BD received a diagnosis of BD‐NOS, which may affect interpretation of our findings when considering potential subsequent transition to other BD subtypes or disorders. However, BD‐NOS deserves inclusion given its preponderance in oBD, demonstrated impairment, and common transition to BD‐I or BD‐II (Axelson et al., 2011; Birmaher et al., 2021; Nurnberger et al., 2011).

## CONCLUSION

Family dynamics are heterogeneous among youth at familial risk for BD (Stapp et al., 2019; Stapp, Mendelson et al., 2020). These data, from a well‐characterized, international high‐risk sample, suggest different ways of crossing the liability threshold to develop early‐onset BD consistent with a multifactorial liability threshold model—conflicted FE and lower BD‐PRS were associated with significantly higher liability for BD compared to well‐functioning FE. Notably, European‐ancestry youth with BD and high‐conflict‐FE/low‐BD‐PRS had increased history of suicidality compared to well‐functioning FE. While much previous work has focused on maltreatment, and others have demonstrated different patterns of illness course linked to BD‐PRS by maltreatment interaction (Aas et al., 2020), we capture FE more broadly and are the first to present the differential effect of BD‐PRS on liability for BD in the high‐risk context depending on FE. These results support focusing on modifiable domains of FE, such as reducing communication conflict and improving family cohesion and adaptability, with the goal of contributing to adolescents' resilience and reducing burden associated with BD.

## AUTHOR CONTRIBUTIONS

**Emma K. Stapp**: Conceptualization; Data curation; Formal analysis; Investigation; Methodology; Project administration; Visualization; Writing – original draft; Writing – review & editing. **Janice M. Fullerton**: Funding acquisition; Methodology; Resources; Writing – review & editing. **Rashelle J. Musci**: Methodology; Supervision; Visualization. **Peter P. Zandi**: Conceptualization; Funding acquisition; Supervision; Writing – review & editing. **Melvin G. McInnis**: Conceptualization; Funding acquisition; Investigation; Resources; Writing – review & editing. **Philip B. Mitchell**: Funding acquisition; Investigation; Resources; Writing – review & editing. **Leslie A. Hulvershorn**: Resources; Writing – review & editing. **Neera Ghaziuddin**: Investigation; Writing – review & editing. **Gloria Roberts**: Resources; Writing – review & editing. **Alessandra G. Ferrera**: Investigation; Writing – review & editing. **John I. Nurnberger**: Conceptualization; Data curation; Funding acquisition; Investigation; Resources; Supervision; Writing – review & editing. **Holly C. Wilcox:** Conceptualization; Funding acquisition; Investigation; Resources; Supervision; Writing – review & editing.

## CONFLICT OF INTEREST STATEMENT

John I. Nurnberger, MD, is an investigator for Janssen. Philip Mitchell has received remuneration from Janssen (Australia) and Sanofi (Hangzhou) for advisory board membership or lectures. The remaining authors have declared that they have no competing or potential conflicts of interest.

## ETHICAL CONSIDERATIONS

Institutional Review Boards approved the research at each of the four US sites, and the University of New South Wales Human Research Ethics Committee approved the research at the Australian site. Written informed consent was obtained for all adult participants, or assent with parental consent for participants under age 18 in the US and 17 in Australia, after receiving a complete description of the study.

## Supporting information

Supporting Information S1

## Data Availability

The data that support the findings of this study may be available on request from the senior investigators. The data are not publicly available due to privacy or ethical restrictions.
