# Supplementary material for: Family environment and polygenic risk in the bipolar high‐risk context
Source: JCPP Adv. 2023 Mar 16;3(2):e12143. doi: 10.1002/jcv2.12143 (PMC10292829; doi:10.1002/jcv2.12143)

**Supplementary Online Content**

**Family Environment and Polygenic Risk in the Bipolar High-Risk Context**

Supplemental Methods. Genotype Imputation

Figure S1. Conceptual latent model

Figure S2. Deciles of BD-PRS in European-ancestry offspring, by presence of offspring BD

Figure S3. Distribution of offspring BD-PRS across PCA-derived genetic ancestry categories

Table S1. Mean Standardized Bipolar Polygenic Risk Scores by Parent Group and Offspring Affected Status

Table S2. Family Environment and its Interaction with Bipolar Polygenic Risk Score on Offspring Bipolar Disorder in offspring of diverse ancestry

Figure S4. Estimated probability of Bipolar Disorder diagnosis by zBD-PRS across three latent profiles of family environment in diverse-ancestry offspring

Table S3. Baseline Clinical Features by Exposure in European-Ancestry Offspring with Lifetime Bipolar Disorder

Figure S5. Estimated probability of Bipolar Disorder diagnosis by zBD-PRS across three latent profiles of family environment in European-ancestry offspring, adjusted for age, sex, genetic ancestry, self-reported race, and parental Bipolar Disorder (familial high-risk status)

This supplementary material has been provided by the authors to give readers additional information about their work.

**Supplemental Methods. Genotype Imputation.**

Following genotype filtering using ENIGMA Phase 3 version 5 cookbook (http://enigma.ini.usc.edu/wp-content/uploads/2020/02/ENIGMA-1KGP_p3v5-Cookbook_20170713.pdf), genotype imputation was conducted using the Michigan Imputation Server (v1.0.3), employing the 1000 Genomes Phase 3 version 5 reference panel (specifying EUR population, as ~10% of sample was ‘non-white’) with phasing by Eagle v2.3. Imputed variants underwent additional filtering, where SNPs failing Hardy-Weinberg Equilibrium (p<0.000001), with MAF<0.01 or Rsq<0.8 and strand ambiguous SNPs were removed.

**Figure S1. Conceptual latent model of gene-environment interaction on offspring bipolar disorder**

**Figure S2. Deciles of BD-PRS in European-ancestry offspring, by presence of offspring BD**

**Figure S3. Distribution of offspring BD-PRS across PCA-derived genetic ancestry categories**

Figure S2 Legend: Participants were categorized into five subgroups based on their PCA-derived genetic ancestry: 0=European (n=327), 1=Mixed Asian (n=19), 2=Asian (n=8), 3=Mixed African (n=12), 4=African (n=33). Top row, traditional boxplot without individual datapoints (raw scores on left, standardized scores on right); bottom row, stripplot with individual datapoints jittered and box with IQR and whiskers (raw scores on left, standardized scores on right).

**Table S1. Mean Standardized Bipolar Polygenic Risk Scores by Parent Group and Offspring Affected Status**

|  | **Total Sample**  (n=399) |  | **High-Risk (oBD)**  (n=244) |  | **Controls**  (n=155) |  | ***Row p*** |
| --- | --- | --- | --- | --- | --- | --- | --- |
| **Mean ± SD** | 1.82e-09 ± 1 |  | -0.17017  ± 0.72081 |  | 0.26788  ± 1.2831 |  | 0.281 |
| ***Offspring Affected Status*** |  | ***Col. p*** |  | ***Col. p*** |  | ***Col. p*** |  |
| **BD^a^** | n=48 |  | n=43 |  | n=5 |  |  |
|  | -.2587016 ± .5402526 | 0.525 | -.277584 ± .5606678 | 0.633 | -.0963129 ± .3034015 | <0.001 |  |
| **No BD** | n=325 |  | n=187 |  | n=138 |  |  |
|  | .0221926 ± 1.009986 |  | -.1208047 ± .7688791 |  | .2159643 ± 1.242612 |  |  |
| *by PCA-ancestry* |  |  |  |  |  |  |  |
| BD |  |  |  |  |  |  |  |
|  |  |  | n=40 |  | n=4 |  |  |
| *European* | – |  | -.3884049 ± .1865085 |  | -.2281288 ± .0830691 |  |  |
|  |  |  | n=2 |  | n=0 |  |  |
| *Mixed Asian* | – |  | .3163946 ± .3462559 |  | – |  |  |
|  |  |  | n=0 |  | n=1 |  |  |
| *Asian* | – |  | – |  | † |  |  |
|  |  |  | n=0 |  | n=0 |  |  |
| *Mixed African* | – |  | – |  | – |  |  |
|  |  |  | n=1 |  | n=0 |  |  |
| *African* | – |  | † |  | – |  |  |
| No BD |  |  |  |  |  |  |  |
|  |  |  | n=164 |  | n=98 |  |  |
| *European* | – |  | -.3429861 ± .1802411 |  | -.3595408 ± .2283602 |  |  |
|  |  |  | n=7 |  | n=10 |  |  |
| *Mixed Asian* | – |  | -.1822898 ± .2411513 |  | -.2182901 ± .4130146 |  |  |
|  |  |  | n=0 |  | n=7 |  |  |
| *Asian* | – |  | – |  | .3202434 ± .1228196 |  |  |
|  |  |  | n=10 |  | n=2 |  |  |
| *Mixed African* | – |  | 1.587564 ± .3955563 |  | .4840152 ± .0573329 |  |  |
|  |  |  | n=6 |  | n=21 |  |  |
| *African* | – |  | 3.176604 ± .2687681 |  | 3.048154 ± .3034056 |  |  |

**Note**: BD, bipolar disorder; BD-PRS, mean bipolar polygenic risk scores at p-value threshold <0.03 standardized in diverse ancestry sample; Col., Column; oBD, offspring of a parent with BD i.e., ‘high-risk’. P-values for *overall comparisons* are based on linear regression using generalized estimating equations with robust standard errors accounting for familial clustering, adjusted for genetic ancestry (first two components of continuous PCA-derived genetic ancestry, as well as 5 PCA-derived categories), and *based on complete case analysis* (not everyone with diagnosis information had genotype data).

† for instances of n=1, BD-PRS withheld for privacy.

^a^ Examining BD-PRS as an exposure and BD-I/II *vs* BD-NOS as the outcome yields a p-value of 0.576.

**Table S2. Family Environment and its Interaction with Bipolar Polygenic Risk Score on Offspring Bipolar Disorder in offspring of diverse ancestry**

|  | **Main Effect of FE** | **BD-PRS x FE Interaction** | **Proportion (n) with Diagnosis** |
| --- | --- | --- | --- |
| **Family Environment Profile** | **Model**  **Significance Tests**  Overall *Wald* and Pairwise *z* | |  |
|  | *Wald*= 1.476, p=0.4780 | *Wald*=8.118, **p=0.0173** |  |
| Well-Functioning | – | – | 0.112 (30) |
| Paternal Conflict | *z*=0.567, p=0.243 | *z*=5.013, **p=0.011**^a^ | 0.178 (15) |
| Maternal Conflict | *z*=0.398, p=0.470 | *z*=4.439, p=0.177^a^ | 0.161 (7) |

Note: BD-PRS, bipolar disorder polygenic risk score based on disease associated SNPs from Psychiatric Genomics Consortium Wave 2 (Stahl et al. 2019) at p-value threshold p<0.03, standardized in the full offspring sample; FE, Family Environment. High-risk and control offspring were modeled together and present in all FE profiles. Main effects model adjusted for offspring age, sex, genetic ancestry (first two components of continuous PCA-derived genetic ancestry and 5 PCA-derived categories), and BD-PRS. Interaction model adjusted for offspring age, sex, and genetic ancestry. N’s are estimates based on posterior probabilities, *rounded to the* *nearest whole number* (sample has 52 analyzed offspring with BD).

^a^ Statistical test of the significance of the interaction term of specific FE class with mean BD-PRS.

**Figure S4. Estimated probability of Bipolar Disorder diagnosis by zBD-PRS across three latent profiles of family environment in diverse-ancestry offspring**

Figure note: Directionality between BD-PRS and BD among offspring with well-functioning FE was positive (OR=6.86, 95% CI=1.28-36.80, p=0.025), whereas among diverse-ancestry offspring with paternal-conflict, a 1SD increase in BD-PRS was marginally inversely associated with offspring BD (OR=0.05, 95% CI=0.001-1.43, p=0.079), demonstrating highest risk for BD among those with high-conflict FEs and lower BD-PRS.

**Table S3. Baseline Clinical Features by Exposure in European-Ancestry Offspring with Lifetime Bipolar Disorder**

|  | **Family Environment** | |  | **Common Genetic Burden** | |  | **Combination** | |  | **Isolating** | |  |
| --- | --- | --- | --- | --- | --- | --- | --- | --- | --- | --- | --- | --- |
|  | WF-FE | High Conflict | *p* | Low BD-PRS | High BD-PRS | *p* | WF-FE, High BD-PRS | High Conflict, Low BD-PRS | *p* | High Conflict, High BD-PRS | WF-FE, Low BD-PRS | *p* |
|  | n=26 | n=16 |  | n=25 | n=17 |  | n=13 | n=12 |  | n=4 | n=13 |  |
| **Suicide Ideation**, n (%) | 7 (26.92) | 12 (75.00) | **0.002** | 12 (48.00) | 7 (41.18) | 0.663 | 3 (23.08) | 8 (66.67) | **0.028** | 4 (100) | 4 (30.77) | **0.015** |
| **Suicide Attempt**, n (%) | 4 (15.38) | 6 (37.50) | 0.102 | 9 (36.00) | 1 (5.88) | **0.024** | 0 (0.00) | 5 (41.67) | **0.009** | 1 (25.00) | 4 (30.77) | 0.825 |
|  | n=23 | n=14 |  | n=21 | n=16 |  | n=12 | n=10 |  | n=4 | n=11 |  |
| **SUD**, n (%) | 7 (30.43) | 6 (42.86) | 0.443 | 8 (38.10) | 5 (31.25) | 0.67 | 3 (25.00) | 4 (40.00) | 0.452 | 2 (50.00) | 4 (36.36) | 0.634 |

Note: WF-FE, Well-Functioning Family Environment; SUD, substance use disorder (any DSM-IV alcohol or drug abuse or dependence). P-value from χ^2^ (offspring with BD were unrelated). These descriptive analyses are based on *complete cases* (some offspring from full sample were missing genotyping and/or clinical feature data). Significant findings, though exploratory, were maintained against Benjamini-Hochberg correction.

**Figure S5. Estimated probability of Bipolar Disorder diagnosis by zBD-PRS across three latent profiles of family environment in European-ancestry offspring, adjusted for age, sex, genetic ancestry, self-reported race, and parental Bipolar Disorder (familial high-risk status)**


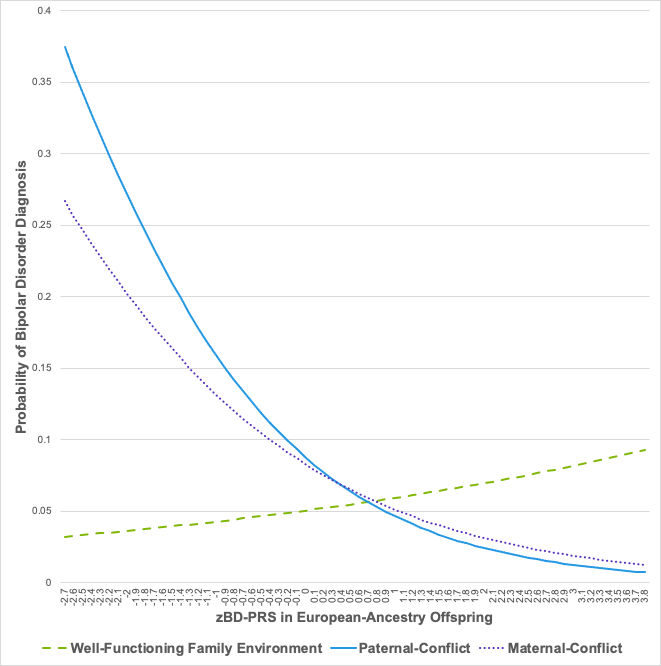

Supplement: Supplementary file 1 — Supporting Information S1 [file JCV2-3-e12143-s001.docx]
